# Plasmonic Micro-Channel Assisted Photonic Crystal Fiber Based Highly Sensitive Sensor for Multi-Analyte Detection

**DOI:** 10.3390/nano12091444

**Published:** 2022-04-23

**Authors:** Q. M. Kamrunnahar, Firoz Haider, Rifat Ahmmed Aoni, Jannatul Robaiat Mou, Shamsuttiyeba Shifa, Feroza Begum, Hairul Azhar Abdul-Rashid, Rajib Ahmed

**Affiliations:** 1 Department of Electronics & Telecommunication Engineering, Rajshahi University of Engineering & Technology, Rajshahi 6204, Bangladesh; konaete30@gmail.com (Q.M.K.); jannatulruet@gmail.com (J.R.M.); 2 Faculty of Engineering, Multimedia University, Cyberjaya 63100, Selangor, Malaysia; firozknu@gmail.com; 3 Integrated Photonics and Applications Centre, School of Engineering, RMIT University, Melbourne, VIC 3001, Australia; 4 Department of Biochemistry and Molecular Biology, University of Dhaka, Dhaka 1000, Bangladesh; shamsuttiyebashifadu.ac.bd@gmail.com; 5 Faculty of Integrated Technologies, Universiti Brunei Darussalam, Gadong BE1410, Brunei; feroza.begum@ubd.edu.bn; 6 School of Medicine, Stanford University, Palo Alto, CA 94304, USA

**Keywords:** surface plasmon resonance, photonic crystal fiber, optical fiber sensors, multi-analyte detection

## Abstract

A dual-channel propagation controlled photonic crystal fiber (PCF)-based plasmonic sensor was presented to detect multiple analytes simultaneously. Plasmonic micro-channels were placed on the outer surface of the PCF, which facilitates an easy sensing mechanism. The sensor was numerically investigated by the finite element method (FEM) with the perfectly matched layer (PML) boundary conditions. The proposed sensor performances were analyzed based on optimized sensor parameters, such as confinement loss, resonance coupling, resolution, sensitivity, and figure of merit (FOM). The proposed sensor showed a maximum wavelength sensitivity (WS) of 25,000 nm/refractive index unit (RIU) with a maximum sensor resolution (SR) of 4.0 × 10^−6^ RIU for channel 2 (Ch-2), and WS of 3000 nm/RIU with SR of 3.33 × 10^−5^ RIU for channel 1 (Ch-1). To the best of our knowledge, the proposed sensor exhibits the highest WS compared with the previously reported multi-analyte based PCF surface plasmon resonance (SPR) sensors. The proposed sensor could detect the unknown analytes within the refractive index (RI) range of 1.32 to 1.39 in the visible to near infrared region (550 to 1300 nm). In addition, the proposed sensor offers the maximum Figure of Merit (FOM) of 150 and 500 RIU^−1^ with the limit of detection (LOD) of 1.11 × 10^−8^ RIU^2^/nm and 1.6 × 10^−10^ RIU^2^/nm for Ch-1 and Ch-2, respectively. Due to its highly sensitive nature, the proposed multi-analyte PCF SPR sensor could be a prominent candidate in the field of biosensing to detect biomolecule interactions and chemical sensing.

## 1. Introduction

In the current COVID-19 pandemic, a highly sensitive, fast response, avoiding false positive responses, and cost-effective biosensor devices are highly desirable [1,2,3,4]. Generally, a sensor is an analytical device which is used to sense unknown analytes from the surrounding medium [5,6]. A PCF-based SPR sensor for multi-analyte detection can be a promising candidate due to its highly sensitive nature to small changes of target analytes. Among the various types of sensors (such as electrochemical, piezoelectric, etc.), optical sensors are the most preferred due to their quick response [7,8], lightweight nature [9], remote sensing abilities, and maximum signal-to-noise characteristics [10,11]. There are several optical sensing techniques, such as micro-ring resonator, resonant mirror, fiber brag grating, photoluminescence, and so on [12,13,14]. SPR is the most effective sensing method due to its label-free detection [15] and wide applications in environment monitoring, chemical sensing, medical diagnosis, telemedicine, virus detections, and so on [16,17]. The SPR phenomenon occurs when the p-polarized light hits the metal surface or at a specific angle of incidence light or with a specific coupling condition [9]. Prism coupling-based SPR sensors (Kretschmann Raether configuration) were very popular in the past for their good sensing performances [18]. However, prism coupling-based SPR sensors are bulky, costly, and have a non-portable structure that limits the sensing performance. Besides, PCF-based SPR sensors solved these issues by miniaturization and remote sensing [13]. PCF is a single-material fiber where air-holes are arranged in various forms (such as hexagonal, octagonal, circular, decagonal, and even in free style). By altering the air-hole arrangement, the sensing performance can be improved. The PCF-based SPR sensor combines the PCF technology with plasmonic science [19], which was initially introduced by Hassani et al. [20]. This combination leads the promising PCF-based SPR sensing technique for a broad range of applications [21]. PCF-based SPR sensors are mainly distributed as internally coated and externally coated according to the plasmonic metal layer position [22]. In the internal sensing method, there exists an option to choose the air-holes which can be filled [23]. Thus, the sensitivity can be improved by selecting the channel location and quantity of the analyte. Fan et al. [24] proposed an analyte filled PCF-SPR sensor with gold and silver layers. The numerical analysis of the structure showed that the maximum WS could reach up to 7040 nm/RIU and 7017 nm/RIU within the RI range of 1.40 to 1.42. Liu et al. [22] proposed another square lattice structure where left and right channels were internally coated with gold. This sensor worked in a broad detection range (1 to 1.43) with the maximum WS of 6300 nm/RIU. In a fabrication point of view, depositing a uniform plasmon layer over a micrometer-scale air-hole surface is quite challenging. Moreover, the filling and cleaning of samples in the tiny air-holes is quite difficult [25].

Through locating the detection layer at the outer surface, the external sensing technique overcomes these challenges. Liu et al. [26] proposed an externally coated, simple circular lattice structure that offered maximum WS of 35,000 nm/RIU with the Amplitude Sensitivity (AS) of 1120 RIU^−1^ in the analyte RI range of 1.34 to 1.40. Additionally, Jiao et al. [27] proposed a dual-core PCF based SPR sensor for external sensing. This sensor obtained WS up to 7600 nm/RIU with a very low detection range. Various modified structures like U-shaped [28], H-shaped [29], V-shaped [30], D-shaped [31,32], slotted microfluidic [33], dual-side polished [34], and so on have been proposed to enhance the performance. Among all, the D-shaped structure improved the performance notably. Liu et al. [35] proposed a D-shaped PCF-SPR sensor where Indium tin oxide (ITO) was used as a plasmonic material. This sensor offered the maximum WS of 15,000 nm/RIU but very poor AS of 442 RIU^−1^. Recently, Firoz et al. [36] proposed a modified D-shaped structure with very promising sensitivity. However, precise polishing and spilling specifications make this type of sensor very complex in practice [34]. Single-analyte detection processes provide promising performance; however, avoiding false positive responses, detection time, and cost by simultaneous detection pursue the idea of the multi-analyte sensing approach [19,37]. The micro-structured optical fiber-based multichannel plasmonic sensor was first proposed by Zheng et al. [38] in 2011. This modified wagon wheel fiber sensor provided a maximum WS of 1535 nm/RIU for channel 1 and 1550 nm/RIU for channels 2 and 3. Since then, various multichannel designs have been proposed by the researchers. Otupuri et al. [33] suggested another multi-channel metal–organic framework (MOF)-based plasmonic biosensor where four elliptical air-holes were arranged in the cladding region. The obtained maximum WS were up to 4600 nm/RIU and 2300 nm/RIU for channel 2 and channel 1 respectively within the RI range of 1.33 to 1.36. Hameed et al. [39] also demonstrated a multi-channel biosensor where gold was coated over the silver layer to protect it from oxidization. The proposed sensor exhibited a maximum WS of 7500 nm/RIU and 4500 nm/RIU for $H_{11}^{x}$ and $H_{11}^{y}$ modes, respectively, with the RI detection range of 1.33 to 1.35. Recently, Bing et al. [40] reported a highly sensitive dual-channel PCF-SPR sensor where a horizontal polishing effort was required according to the structure. The maximum WS could reach 11,600 nm/RIU and 10,600 nm/RIU for two channels respectively with the RI range of 1.33 to 1.40. Yasli et al. [19] also numerically investigated a multi-channel PCF sensor where the structure relies on four concentric analyte channels. According to the spectral sensitivity analysis, this unique sensor could offer the maximum WS of 2500 nm/RIU and 3083 nm/RIU with a maximum resolution of 4 × 10^−5^ RIU^−1^ and 3.2 × 10^−5^ RIU^−1^ for channel 1 (x-polarized) and channel 2 (y-polarized), respectively, in the RI range of 1.33 to 1.366. Very recently, Bing et al. [41] suggested an up-core PCF for double sample synchronous detection. In this structure, one analyte is placed in the central air hole and the other in the outer surface. All these unique-featured multi-channel sensors can be applicable in various fields, but the performances are not remarkable. Therefore, a highly sensitive sensor is required to increase the sensing accuracy while minimizing the design complexity. Additionally, the reported sensor structures are very complex in terms of fabrication.

Here, we proposed a simple, highly sensitive, multi-channel-based PCF SPR sensor which works in a wide sensing region (visible to near-infrared). To enhance the light-mater interactions between the core-guided mode and the surface plasmon polariton (SPP) mode, optimized air holes were used, which control the light-guiding direction of the proposed PCF. Due to the outer surface of the plasmonic gold layer, the proposed sensor will be able to sense the unknown analyte based on an external sensing approach. Considering the fabrication point of view, we extensively investigated the tolerance of the key geometrical parameters.

## 2. Structural Design and Numerical Analysis

**Figure 1:**
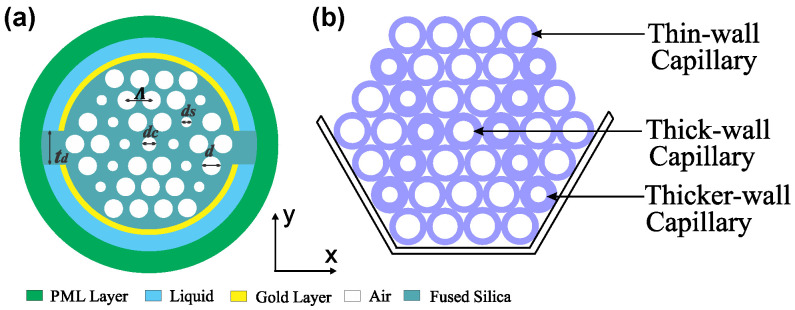
(**a**) 2D cross-sectional view of the proposed sensor, and (**b**) stack preform.

The modelling and the numerical analysis of the proposed sensor were accomplished by the Finite Element Method-based mode solver, commercial COMSOL Multiphysics software. The two-dimensional cross-sectional image of the designed sensor is represented in Figure 1a. The stacked preform of the proposed fiber is shown in Figure 1b. The arrangement here relies on two distinct air-holes grouped in three hexagonal air-hole rings, along with the central air hole. To reduce the effective refractive index, the center air-hole with diameter *d_c_* was used and thus it facilitated the phase-matching properties between the plasmonic modes and the core-guided mode. The regular air-hole (diameter, *d*) allows the concentration of the energy in the fiber core. The scaled down air holes (diameter, *d_s_*) control the direction of light propagation through the PCF and help to stimulate the surface electrons by accumulating the evanescent field. All the air-holes were positioned in a uniform center-to-center distance known as pitch (*Λ*). 

**Figure 2:**
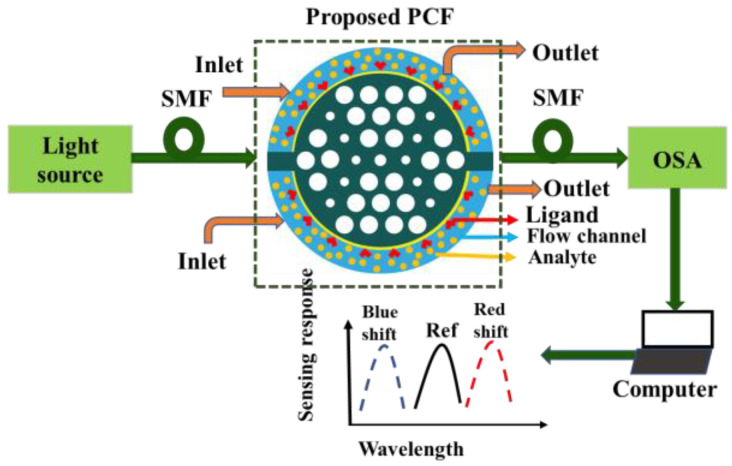
The typical sensing mechanism of the proposed sensor.

In the design, two channels with uniform thickness (*t_a_*) were adopted to hold the analytes. Here the upper channel is defined as channel-1 (Ch-1) and the lower channel is defined as channel-2 (Ch-2). The reliable gap between two channels is denoted as *t_d_* for avoiding the overlap of light coupling. In both channels, noble metal gold (thickness, *t*) was coated internally to create SPR effects. The analyte channels were placed at the outer surface of the PCF to make the cleansing and filling process easier, which is the main drawback of internal coating structure. To absorb the scattering radiation, the boundary condition perfectly matched layer was used at the outer surface. Though PML has no existence in practice, it was used to make the computation region finite. We optimized the structural parameters (*d_c_, d, d_s_, Λ,* and *t*) to operate the sensor in optimal conditions. We selected the values of *Λ =* 1.65 µm, *d_c_* = 0.3*Λ*, *d* = 0.7*Λ*, *d_s_* = 0.2*Λ*, *t* = 40 nm as optimal after careful investigations. In the Figure 1b, regular air holes (diameter *d*) are represented by thin-wall capillary. Similarly scaled down air holes (diameter *d_s_*) and center air-holes (diameter *d_c_*) are presented as thick and thicker-wall capillaries, respectively. We chose each parameter by performing several simulations while keeping other values constant. For performance accuracy, extremely fine mesh elements were used in the numerical analysis. Here, the mesh properties are total number of triangular elements, edge elements, and the vertex elements are 39,116, 2630, and 172 respectively, average element quality is 0.9392, minimum element quality is 0.6511, and the mesh area is 231.2 µm^2^. Fabrication of the proposed sensor is very straight forward where commercially available capillary tubes such as 19/25, 18/20, and 16/20 mm (inner to outer diameter) can be used to make the *d_c_, d, d_s_* of the proposed sensor to reduce the fabrication cost [12]. Four rings of capillaries will be stacked into a 2-mm diameter cane to make a preform of the proposed sensor. Therefore, by controlling the temperature and pressure in the fiber drawing process, we obtain the exact shape of the proposed sensor. After the fiber fabrication, the main difficult task is the accurate placement of the Au layer on the surface of channels. The atomic layer deposition (ALD) or chemical vapor deposition (CVD) methods can be used to place the layer of gold [42,43]. Later, two channels will be placed on the plasmonic layer by considering a distance of *t_d_* = 1 µm. Under the vacuum pressure, the jacket is collapsed with the proposed PCF structure. The background material silica is characterized by the Sellmeier equation [34]:

(1) $$n^{2}\left(\lambda \right)=1+\frac{B_{1}\lambda^{2}}{\lambda^{2}-C_{1}}+\frac{B_{2}\lambda^{2}}{\lambda^{2}-C_{2}}+\frac{B_{3}\lambda^{2}}{\lambda^{2}-C_{3\;}}$$

where, *n* is the wavelength-dependent refractive index of the fused silica, λ is the wavelength in µm, *B_1_, B_2_, B_3_, C_1_, C_2_,* and *C_3_* are the Sellmeier constants. The values of these constants are 0.69616300, 0.407942600, 0.897479400, 0.00467914826 µm^2^, 0.0135120631 µm^2^, and 97.9340025 µm^2^, respectively. We emphasize the fact that this equation is applicable for the wavelength range of 0.22 to 3.71 µm [9]. The RIs of fused silica varies very little with temperature, being around 1.28 × 10^−5^ (per degree Celsius) only [44]. Plasmonic metal has a strong impact on the SPR phenomenon. Silver (Ag), gold (Au), copper (Cu), aluminum (Al), indium tin oxide (ITO), etc. are the most reliable plasmonic materials in the optical frequency range [33]. Gold (Au) and silver (Ag) are the most popular among all as they offer low damping loss in the visible to near-infrared region. Though Ag provides a sharper resonance peak and is free from inter-band transitions in the visible optical region, it experiences oxidization in steamy environments [41]. However, gold is the best choice in nanoscale plasmonics as it is chemically inert, bio-compatible, easily functionalized, and provides a massive shift in the resonance wavelength. The plasmonic material gold RI is obtained by the Drude–Lorentz model [28]. Later, by following the relation between the refractive index and dielectric constants, we calculated the real and imaginary refractive index of gold.

(2) $$\varepsilon_{Au}=\varepsilon_{\alpha }-\frac{\omega_{D}^{2}}{\omega \left(\omega +j\gamma_{D}\right)}-\frac{\Delta \varepsilon \Omega_{L}^{2}}{\left(\omega^{2}-\Omega_{L}^{2}\right)+j\Gamma_{L}\omega }$$

where, *ε_AU_* denotes the permittivity of gold, *ε_∞_* = 5.9673 is the permittivity at high frequency, angular frequency is denoted by *ω*, *ω_D_* is the plasma frequency, *γ_D_* is the damping frequency, where *ω = 2πc/λ*, *ω_D_* = 4227.2π THz, *γ_D_* = 31.84π THz and weighting factor Δ*ε* = 1.09. The spectral width *Γ_L_* = 209.72π THz and oscillator strength *Ω_L_* =1300.14π THz, respectively. Figure 2 shows the typical set-up for the sensing process of the proposed sensor. Firstly, the incident light was launched from the optical tunable source. By passing through the polarizer and polarizer controller, it turned into a linearly polarized light. The linearly polarized light was introduced into the proposed sensor via the single-mode fiber (SMF) as the sensor is small. The coupling between the SMF and the proposed sensor was obtained by splicing techniques. The inlets and outlets of the analyte samples were maintained by a continuous pump. When the analyte sample and ligands interacted with each other, the effective refractive index changed, and the wavelength shifted either in shorter wavelengths or the longer wavelengths. An optical spectrum analyzer (OSA) measured the transmitted power that is connected to the sensor via another SMF. Finally, the output was measured by the computer connected to the OSA. In the wavelength interrogation method, the performance is measured by observing the resonance wavelength shift. However, in intensity-based analysis, the sensitivity is measured with the change of resonance intensity [19].

## 3. Results and Discussions

**Figure 3:**
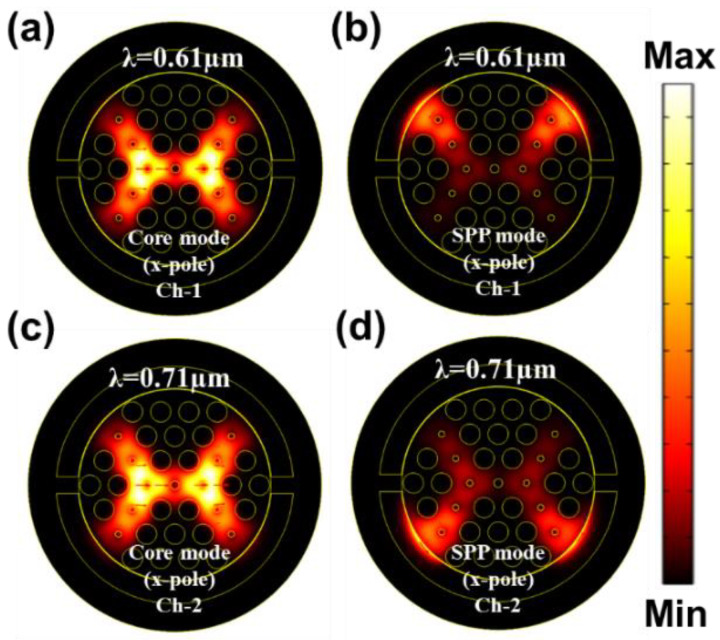
EM field distribution of the core guided mode and the SPP mode for (**a**,**b**) Ch-1 at RI 1.32 and (**c**,**d**) Ch-2 at RI 1.36.

**Figure 4:**
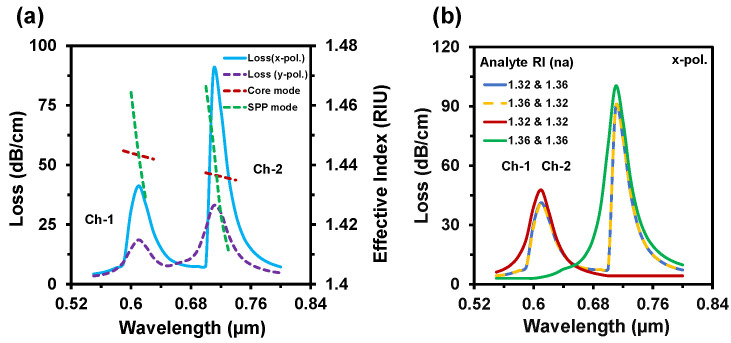
(**a**) The dispersion relationship of the proposed sensor for n_a_ = 1.32 & 1.36 and (**b**) analyte channels dependency behavior at analyte RI of 1.32 and 1.36.

The guided evanescent field which is generated during the penetration of core light is the fundamental requirement of the SPR phenomenon. A surface plasmon wave (SPW) is generated when the evanescent field excites the electrons in the metal layer by hitting [18]. Resonance occurs when the frequency of free electrons and the incident photons are identical. At this condition, maximum energy transfers from the core guided mode to the SPP mode. SPW is very sensible to the surrounding medium RI (SRI). It can detect any slight change of SRI and the resonance peak shifts with this change. Thus, it can detect any unknown analyte. Figure 3a–d represent the electric field distribution of the core guided mode and the SPP mode for Ch-1 and Ch-2 respectively. Figure 4a depicts the dispersion relationship of the core-guided mode, surface plasmon polariton (SPP) mode, and the confinement loss as a function of wavelength for dielectric RI 1.32 and 1.36 at Ch-1 and Ch-2, respectively. Phase matching occurred at 0.61 µm and at 0.71 µm for two channels separately. At these points, the real effective part of both modes (core-guided mode and SPP mode) intersected with each other. Maximum confinement loss (CL) appeared at this point. According to Figure 4a, the x-polarized mode provided a sharper resonance peak than the y-polarized mode. So, the x-polarized mode was considered for the entire analysis. CL plays a critical role to evaluate other sensing performances, which is calculated from the following equation [30]:

(3) $$\alpha \left(\mathrm{dB}/\mathrm{cm}\right)=8.686\times k_{0}Im\left(n_{eff}\right)\times 10^{4}$$

where *K_0_* = 2π/λ is the wave number in free space and *Im(n_eff_*) is the imaginary part of the effective refractive index. 

Figure 4b represents the sensing channels’ dependency on the analyte RI variation. To investigate the channel characteristics, firstly we set RI 1.32 for Ch-1 and RI 1.36 for Ch-2. 

**Figure 5:**
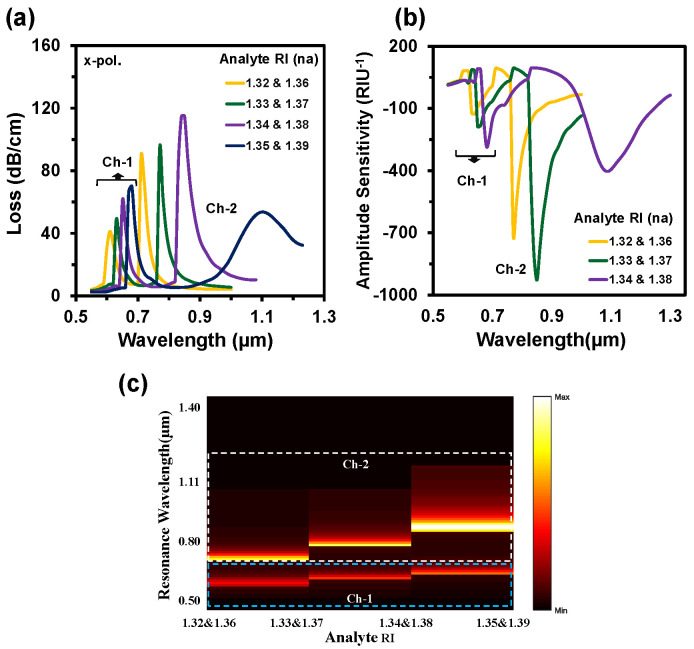
(**a**) CL spectra, (**b**) Amplitude sensitivity, and (**c**) normalized 2D map of CL intensity of the proposed sensor when the analyte RI of Ch-1 and Ch-2 varies simultaneously.

The proposed sensor showed resonant wavelengths at 610 nm and 710 nm, respectively, for Ch-1 and Ch-2. Additionally, the sensor showed the same spectrum with the same resonant wavelength while switching the analyte to RI 1.36 for Ch-1 and RI 1.32 for Ch-2. To further investigate the channel effect, we infiltrated the same analyte RI in both channels. In this case, we also observed that the resonance wavelength occurred at 610 nm for RI 1.32 and 710 nm for RI 1.36. The confinement losses slightly increased, as the maximum incident light was absorbed by a single analyte (e.g., analyte RI 1.32 or 1.36). This happened due to the confinement loss for a certain RI fully relying on the coupling strength between core-guided mode and SPP mode. This strong coupling depends on the refractive index contrast between fiber-core and analyte RI. The refractive index contrast is high at lower analyte RI, resulting in light confines in the core and leading to low confinement loss. Besides, the refractive index contrast decreased with the increase of analyte RI; as a result, core light coupled more at the metal surface, which led to the higher confinement loss. From the above discussion, we can anticipate that the proposed sensor’s performance is independent to the sensing channels. Figure 5a depicts the CL spectra of the reported sensor when analyte RIs varied simultaneously in both channels. With any tiny change of analyte RI (n_a_), the effective RI contrast between the plasmonic mode and the core-guided mode reduced [45]. As a result, more evanescent light passed and coupling intensity increased. So, the CL increases with the increment of analyte RI and the resonance peak shifts towards the longer wavelengths. For demonstrating the multi-analyte phenomena of the proposed sensor, sensing RIs range was divided into two groups: Ch-1 and Ch-2 (see Figure 5a). In Ch-1, the analyte RI varied from 1.32 up to 1.35. Besides, the analyte RI varied from 1.36 up to 1.39 for Ch-2. That means Ch-1 is used for detecting lower RIs and Ch-2 for the higher RIs. For Ch-1 the maximum loss was 70 dB/cm and for Ch-2 it was around 132 dB/cm. The resonance peak appeared at 0.61 µm, 0.63 µm, 0.65 µm, and 0.68 µm for the analyte RIs of Ch-1 and 0.71 µm, 0.77 µm, 0.84 µm, and 1.10 µm for the analyte RIs of Ch-2, respectively. In addition, the loss spectrum decreased for the analyte RI of 1.39 because of the lower value of the effective index (fundamental mode) compared with the respective analyte RI [44]. The sensing performance of a sensor can be measured by two different methods. One is the wavelength interrogation (WI) method, and the other is the amplitude interrogation (AI) method. In the WI method, the WS is calculated by observing the resonance wavelength shift. The WS of a sensor is obtained from [34]:

(4) $$S_{\lambda }\left(\lambda \right)=\Delta \lambda_{peak}/\Delta n_{a}$$

where, Δ*λ_peak_* denotes the resonant wavelength (RW) shift and Δ*n_a_* is the alteration of analyte RIs. 

The WSs were 2000 nm/RIU, 2000 nm/RIU, and 3000 nm/RIU for RI 1.32, 1.33, and 1.34, respectively at Ch-1 of the proposed sensor with an average WS of 2333 nm/RIU. Similarly, at Ch-2 the obtained WSs were 6000 nm/RIU, 8000 nm/RIU, and 25,000 nm/RIU for RI 1.36, 1.37, and 1.38, respectively. The acquired average WS was 13,000 nm/RIU for Ch-2. This simple hexagonal PCF sensor could detect analyte RIs from 1.32 to 1.39. Numerous biomolecules and biochemicals are within this range, such as intestinal mucosa (human) = 1.329–1.338, urine (human) = 1.3415–1.3464, ethanol = 1.361, commercial hydrocarbon mixtures = 1.366, various cancerous cells = 1.36–1.40, and so on [10,22,35]. The AS method is cost-effective due to it being free from wavelength manipulation as well as the entire wavelength spectra not being required [27]. The AS is calculated from the following equation [21]:

(5) $$S_{A}\left(\lambda \right)\left[RIU^{-1}\right]=-\frac{1}{\alpha \left(\lambda ,n_{a}\right)}\frac{\partial \alpha \left(\lambda ,n_{a}\right)}{\partial n_{a}}$$

where, *α(λ, n_a_)* denotes the CL and *δα(λ, n_a_)* is the loss difference between two consecutive analyte RIs. Figure 5b illustrates the AS spectra of the proposed sensor when the sample RI varied simultaneously in two channels. The maximum AS of Ch-1 was 287 RIU^−1^ and 928 RIU^−1^ for Ch-2, respectively. The normalized 2D map of the CL intensity is visualized in Figure 5c. The coupling intensity increased when the analyte RI increased. Double coupling happened at the same time due to the simultaneous changing of two analyte RIs. In Figure 5c, the lower part presents the coupling intensity of Ch-1 and the upper part for Ch-2. SR is a crucial parameter as it defines the detection capability of any small changes of the dielectric RI. The SR is obtained from [5]:

(6) $$R\left(RIU\right)=\frac{\Delta n_{a}\times \Delta \lambda_{min}}{\Delta \lambda_{peak}}$$

where Δ*λ_min_* denotes the minimum wavelength resolution. By considering Δ*λ_min_* = 0.1 and Δ*n_a_* = 0.01, the maximum sensor resolution is calculated as 3.3 × 10^−5^ RIU and 4.0 × 10^−5^ RIU for Ch-1 and Ch-2, respectively.

**Figure 6:**
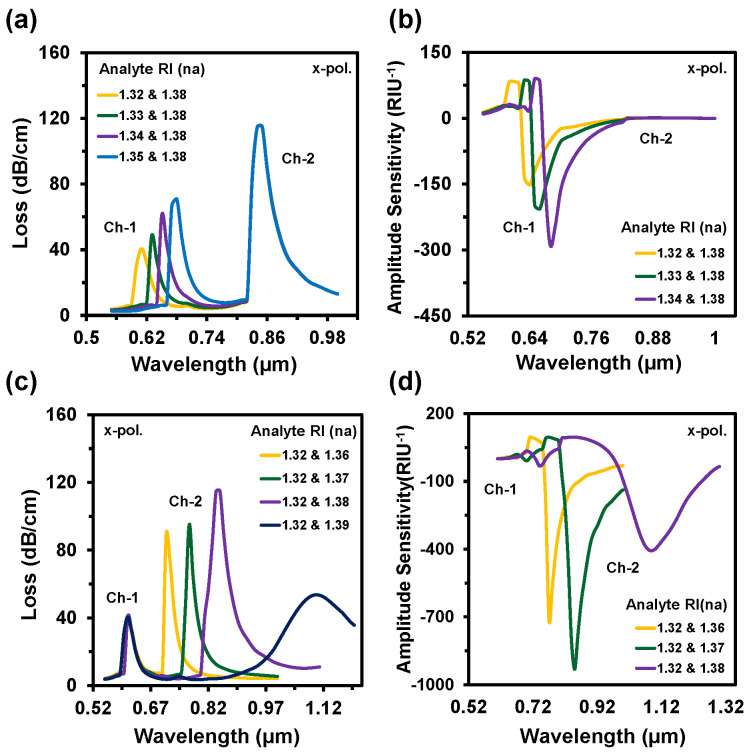
(**a**,**c**) CL spectra and (**b**,**d**) amplitude sensitivity of the proposed sensor when the analyte RIs of Ch-1 varies with constant Ch-2 and Ch-2 varies with fixed Ch-1, respectively.

So, the reported sensor could sense the minimal change up to 10^−6^ order. The obtained LOD was 1.11 × 10^−8^ RIU^2^/nm and 1.6 × 10^−10^ RIU^2^/nm for Ch-1 and Ch-2, respectively. The LOD can be executed by the division of maximum resolution and the sensitivity (*R/S_λ_*) [46]. Figure 6a,b shows the CL spectra and the AS when the analyte RI varied from 1.32 to 1.35 at Ch-1 and RI 1.38 was fixed at Ch-2. At Ch-1, with the increase of analyte RI, the RW shifted towards the longer wavelength. The resonance peak displayed at 0.61 µm, 0.63 µm, 0.65 µm, and 0.68 µm for Ch-1 but the resonance peak stayed fixed at 0.85 µm for Ch-2 as RI was set at 1.38. The maximum WS of 3000 nm/RIU was obtained for Ch-1. Similarly, Figure 6c–d represents the spectrum of CL and AS when the Ch-2 altered from 1.36 to 1.39 and Ch-1 stayed at RI 1.32. The resonance peak appeared at 0.71 µm, 0.77 µm, 0.85 µm, and 1.10 µm for the analyte RIs of 1.36, 1.37, 1.38, and 1.39, respectively. The acquired WSs were 6000 nm/RIU, 8000 nm/RIU, and 25,000 nm/RIU and the ASs were 712 RIU^−1^, 931 RIU^−1^, and 400 RIU^−1^ for RI of 1.36, 1.37, and 1.38 in Ch-2, respectively, but no change was observed in Ch-1 as RI was fixed at RI 1.32.

**Figure 7:**
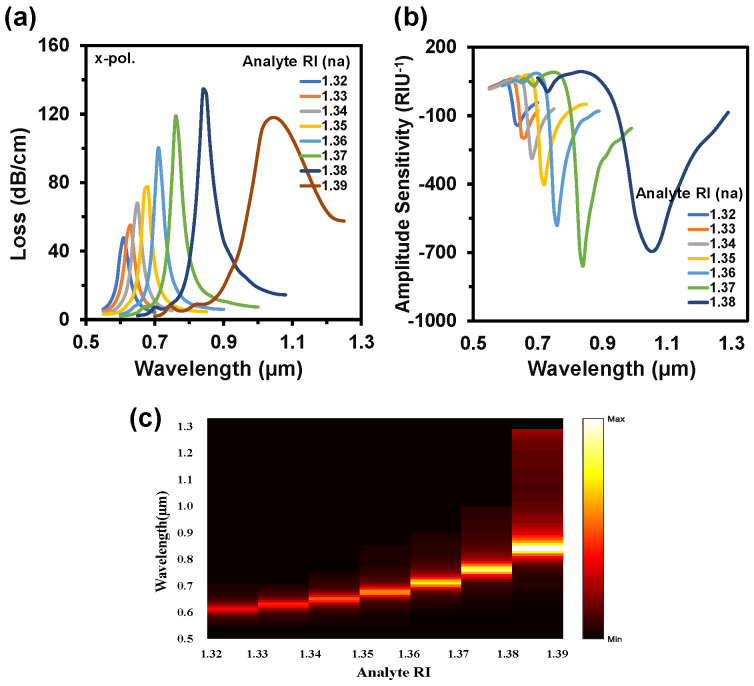
(**a**) CL spectrum while the same analyte RI is infiltrated in both channels, (**b**) AS for the variation of analyte RI from 1.33 to 1.38, and (**c**) normalized 2D map of CL intensity when the same analyte RIs are used in both channels.

**Figure 8:**
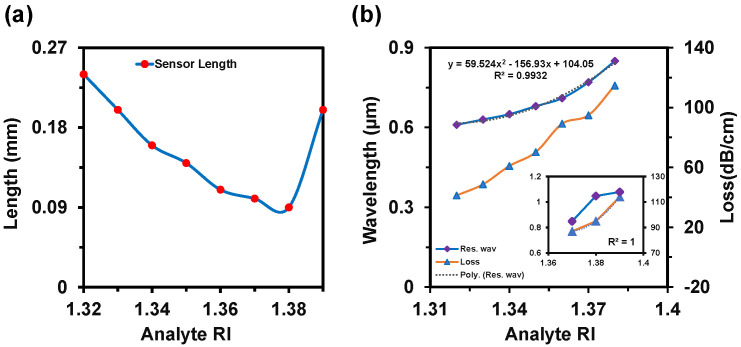
(**a**) Sensor length and (**b**) resonance wavelength shift and loss curve fitting of the proposed sensor as a function of analyte RI from 1.32 to 1.39.

Figure 7a,b depict the CL and AS spectra when both channels used the same analyte RIs. This alteration happened from RI 1.32 to 1.39. In this variation, the sensor exhibited a maximum WS of 20,000 nm/RIU and an AS of 760 RIU^−1^ with an SR of 5 × 10^−6^ RIU^−1^. In this case, the LOD was 3.05 × 10^−6^ RIU^2^/nm. Figure 7c represents the 2D map of CL intensity where minimum intensity was observed at RI 1.32 and the maximum intensity was observed at RI 1.39. Sensor length is very important to determine the feasibility of any sensor, which is illustrated in Figure 8a. This parameter is inversely proportional to the CL. If the loss is too high, then the input light will not be able to reach at the output port. On the contrary, if the loss value is too low, the coupling will be very poor. This type of coupling degrades the sensitivity performances. So, it is important to always focus on acquiring an optimal value for a good sensor. The sensor length can be determined from [42]:

(7) $$L=\frac{1}{\alpha \left(\lambda ,n_{a}\right)}$$

where, *α*(*λ*, *n_a_*) is the confinement loss. For this proposed sensor, the maximum sensor length was obtained as 0.24 cm, which is better than the reported sensors [5]. FOM defines the detection capability of any sensor which is obtained by [12]:

(8) $$FOM=\frac{S_{\lambda }\left(nm/RIU\right)}{FWHM}$$

where, *S_λ_* denotes the WS and FWHM is the full width at half maxima. FOM increases while the FWHM decreases. The FWHMs were 40 nm, 20 nm, 20 nm, and 30 nm for Ch-1 and 30 nm, 20 nm, 50 nm, and 150 nm for Ch-2 when both channel RIs varied simultaneously. So, the desired maximum FOMs were 150 RIU^−1^ and 500 RIU^−1^ for Ch-1 and Ch-2, respectively. The RW and the loss curve fitting of the proposed sensor as a function of analyte RI is presented in Figure 8b. 

The polynomial fitting was obtained as R^2^ = 0.9932. The value of R^2^ was very close to unity, which means that the sensor is highly sensitive. The sensor performances are summarized in detail in Table 1. Additionally, we show the relation between the analyte RI and propagation loss at the resonant wavelength in Figure 8b. This relation helps to corelate the coupling strength as a function of analyte RI. Moreover, if the loss is very high, then the practical realization of that sensor is not possible as the input signal will disappear immediately and will not be able to produce a measurable signal at the output side [12,47].

## 4. Fabrication Tolerances

**Figure 9:**
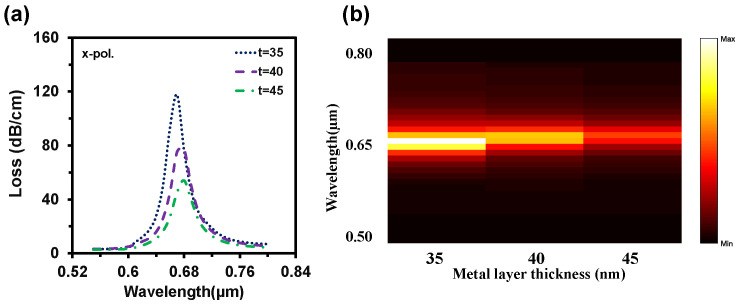
Impact of gold layer thickness variation (*t* = 35 nm, 40 nm, and 45 nm) on (**a**) CL spectra and (**b**) light sensitivity alteration with the change of *t* when RI 1.35 is used in both channels.

In terms of practical realization, it is quite challenging to fabricate a sensor with the exact parameter values. For this reason, ±2% variations from the optimum values were generally observed to investigate the tolerances [43]. However, we considered ±10% variations for each structural parameter (*Λ, d_c_, d, d_s_*) to show the feasibility of the proposed sensor. At first, we optimized the metal layer thickness as the SPP wave generates through the metal layer, and the plasmonic layer thickness variation has a strong impact on sensing performances. Figure 9a represents the impact of gold layer thickness variations (35 nm, 40 nm, and 45 nm) on CL spectra. The loss depth rose when the gold layer thickness scaled up. It is clear from Figure 9a that RW red shift happened when the gold layer became thicker, but loss depth reduced. This happens due to the damping properties of gold. Figure 9b symbolizes the normalized 2D map of the CL intensity when the plasmon layer varied (like t = 35 nm, 40 nm, and 45 nm). For 35 nm, strong coupling occurred, and it weakened with the increment of the thickness. 

**Figure 10:**
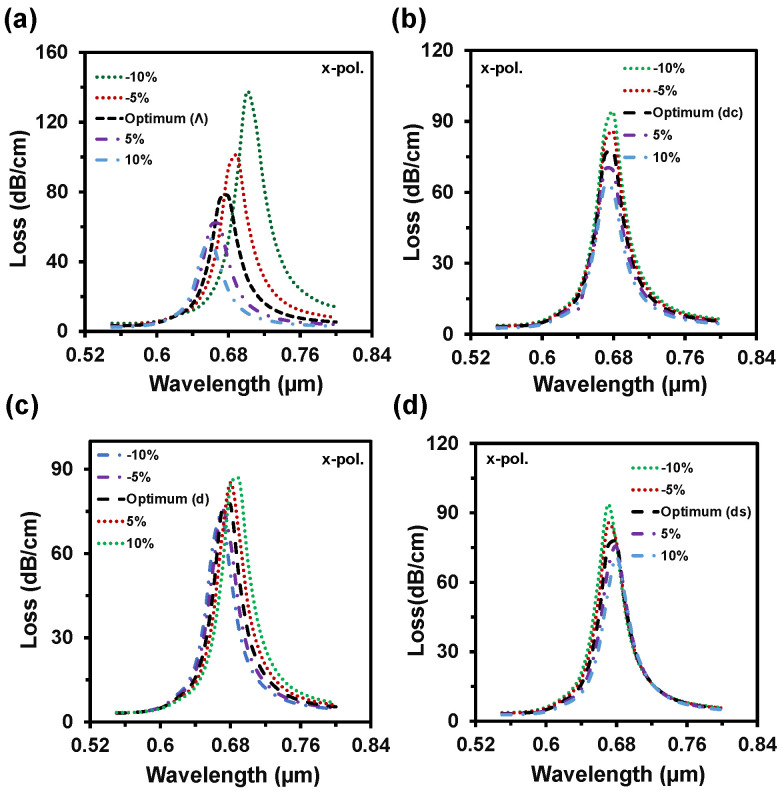
CL spectra with the structural parameter variation of (**a**) pitch, Λ, (**b**–**d**) air-hole diameters dc, d, ds up to ±10% from the optimum value when same analyte RI 1.35 used in both channels.

By considering the performance, we choose 40 nm as an optimal value. Figure 10a depicts the impacts of pitch value variations on CL. Here, the loss depth increased with the decrement of pitch value and the RW shifted from 0.68 µm to 0.69 µm and 0.7 µm with these changes. On the contrary, loss depth reduced to 61 dB/cm and 53 dB/cm when the pitch value increased by 5% to 10% from neutral. Moreover, The RW fluctuated 0.68 µm to 0.67 µm and 0.66 µm with this increment of the pitch value. Similarly, loss depth increased with the scaling down of the center air-hole diameter or vice-versa (from Figure 10b). Though the loss peak fluctuated, there was no significant change in RW. So, the WS remained the same. With the enlargement of the regular air-hole diameter, the effective index of core-guided mode decreased, resulting in the loss peak rising and redshift occurrence in RW. On the other hand, loss depth decreased with the scaling down of *d* and the RW faced the blue shift. All these are evident from Figure 10c. Small air-hole diameter has a strong influence in phase matching. 

From Figure 10d, the loss depth rose and blue shift happened with the cutback of the diameter (*d_s_*). The opposite scenario is created by scaling up the small air-hole diameter. According to the fabrication tolerance investigation, it is visible that even if the parameters fluctuate up to ±10%, it will not the hamper the sensing performances. Table 2 shows a performance comparison between the proposed sensor and the existing reported sensors. It seems that performance of the proposed sensor is extremely high compared with other reported multianalyte detection-based PCF-SPR sensors. From Table 2, most of the reported sensors have only investigated the sensing performance in term of wavelength interrogation method. Besides, the proposed sensor was investigated by wavelength and amplitude interrogation methods. Additionally, we figured out all the sensing performance (see Table 1) considering all the important performance measurement parameters, which bring out the sensing accuracy.

## 5. Conclusions

A simple propagation-controlled dual-channel PCF SPR sensor was numerically investigated for the simultaneous detection of multiple analytes. All the structural parameters were investigated in detail with ±10% fabrication tolerance for optimal sensing performance. The proposed sensor exhibited the maximum WS of 3000 nm/RIU and 25,000 nm/RIU for Ch-1 and Ch-2, respectively, in the RI range of 1.32 to 1.39. The maximum amplitude sensitivity was obtained as 284 RIU^−1^ and 928 RIU^−1^ for Ch-1 and Ch-2, respectively. The proposed sensor can detect the change of analyte RIs around 10^−6^ order. Additionally, the FOM of the proposed sensor was 150 and 500 RIU^−1^ for Ch-1 and Ch-2, respectively. The proposed sensor is not limited for multi-analyte detection; it can be applied for single analyte detection as well. Multi-analyte detection-based sensors also offer cost-effective and fast detection capability that improves sensing technology tremendously. Due to its simple structure with high sensing performance, the proposed sensor will be a potential candidate for bimolecular and chemical detection.

## Figures and Tables

**Table 1:** Performance overview of the proposed multi-channel PCF-based SPR sensor.

Channel	Analyte RI	Res. Peak Wave. (nm)	Res. Peak Shift (nm)	Wavelength Sensitivity (nm/RIU)	Average WS (nm/RIU)	Wavelength Resolution (RIU)	FWHM (nm)	Amp. Sen. (RIU^−1^)	FOM (RIU^−1^)
	1.32	610	20	2000		5.0 × 10^−5^	40	126	50
	1.33	630	20	2000	2333	5.0 × 10^−5^	20	187	100
Ch-1	1.34	650	30	3000		3.3 × 10^−5^	20	287	150
	1.35	680	N/A	N/A		N/A	30	N/A	N/A
	1.36	710	60	6000		1.6 × 10^−5^	30	711	166
Ch-2	1.37	770	80	8000	13,000	1.25 × 10^−5^	20	928	400
	1.38	850	250	25,000		4.0 × 10^−6^	50	395	500
	1.39	1100	N/A	N/A		N/A	150	N/A	N/A

**Table 2:** Performance comparison of the proposed multi-channel PCF sensor with the reported sensors.

Ref	Structure Type	Sensing Range	Wave.Sen. (nm/RIU)	Wav.Res. (RIU)	Amp. Sen. (RIU^−1^)	FOM (RIU^−1^)
[19]	Multi-channel PCF-based SPR sensor.	1.33–1.366	2500 (ch-1) 3083 (ch-2)	4.0 × 10^−5^ 3.2 × 10^−5^	N/A	N/A
[33]	Multi-channel SPR biosensor	1.33–1.36	4600 (ch-1) 2300 (ch-2)	2.0 × 10^−5^ 7.0 × 10^−5^	425 131	N/A
[38]	Modified fiber sensor for multi-analyte sensing	1.33–1.46	1535 (ch-1) 1550 (ch-2)	6.51 × 10^−6^ 6.45 × 10^−6^	N/A	N/A
[39]	Two-channel PCF-based SPR biosensor	1.33–1.35	7500 (ch-1) 2500 (ch-2)	2.1 × 10^−5^ 2.33 × 10^−5^	N/A	N/A
[40]	Dual-channel PCF-SPR sensor	1.34–1.40	11,600 (ch-1) 10,600 (ch-2)	8.62 × 10^−6^ 9.71 × 10^−6^	N/A	N/A
[41]	Double sample detection sensor	1.34–1.39	8300	1.20 × 10^−6^	N/A	N/A
[46]	Multi-analyte sensing plasmonic sensor	1.33–1.37	3600 (ch-1) 3700 (ch-2)	N/A	N/A	N/A
[47]	Hollow dual-core PCF-SPR sensor	1.36–1.41	23,000 (ch-1) 30,000 (ch-2)	3.33 × 10^−6^	N/A	N/A
This work	PCF based plasmonic sensor for multi-analyte detection	1.32–1.39	3000 (ch-1) 25,000 (ch-2)	3.33 × 10^−5^ 4.0 × 10^−6^	287 928	150 500

## Data Availability

Data sharing is not applicable to this article.
